# Light-regulated chloroplast morphodynamics in a single-celled dinoflagellate

**DOI:** 10.1073/pnas.2411725121

**Published:** 2024-11-15

**Authors:** Nico Schramma, Gloria Casas Canales, Maziyar Jalaal

**Affiliations:** ^a^Van der Waals-Zeeman Institute, Institute of Physics, University of Amsterdam, Amsterdam 1098XH, The Netherlands; ^b^Institute for Biodiversity and Ecosystem Dynamics, University of Amsterdam, Amsterdam 1098XH, The Netherlands

**Keywords:** chloroplast network, active matter, morphodynamics, organelle movement, photoadaptation

## Abstract

Photosynthetic algae play a significant role in oceanic carbon capture. However, their performance is constantly challenged by fluctuations in environmental light conditions. While phototaxis is a common strategy to cope with such fluctuations, nonmotile species must adopt alternative mechanisms to avoid light-induced damage. Here, we show that the nonmotile, single-celled marine dinoflagellate *Pyrocystis lunula* contains a chloroplast network that undergoes strong deformation in response to strong light. By exposing cells to various physiologically relevant light conditions and applying temporal illumination sequences, we find that the light-induced network morphodynamics follows dynamic rules similar to temporal low-pass filtering. We develop a mathematical formalism to model the light-regulated behavior, exposing the relevant timescales of the morphodynamic response. Moreover, confocal microscopy reveals that the unusual reticulated morphology exhibits properties similar to auxetic metamaterials, facilitating the rapid and drastic deformation necessary for the light-avoidance motion, confined by the cell wall. This mechanism reduces the effective chloroplast area under high light conditions, minimizing light absorption and preventing photodamage. Our findings demonstrate that the intricate connection between the chloroplasts topologically complex structure and active dynamics enables the dinoflagellate’s dynamic adaptation to changing light environments, thereby supporting essential life-sustaining processes.

## Significance

How do nonmotile organisms protect themselves from intense light? While motile single-celled algae can swim toward or away from light (phototaxis), many photosynthetic algae are nonmotile. Studying the nonmotile dinoflagellate *Pyrocystis lunula*, we reveal the dynamics of a complex chloroplast network that undergoes rapid intracellular morphological reorganization in response to changing light. We found that the topologically complex network structure facilitates strong intracellular deformation under cell wall confinement. The cell’s response resembles a low-pass filter for light-fluctuations, distinguishing between irrelevant and relevant light-stimuli. These results illustrate an elegant adaptation process at a single-cell level toward environmental fluctuations. Besides the physiological and ecological relevance, the light-regulated morphodynamics of the chloroplast network represents a well-controlled active matter system, manifesting a biological metamaterial.

Light is fundamental for life, yet excessive exposure can be detrimental to biological processes. Photosynthetic organisms have developed multiple strategies across scales to adapt to changing light conditions, from molecular adaptation responses such as nonphotochemical quenching (NPQ) to the biased growth toward light (phototropism) on organismal scale (1–3). Adaptation to strong light in the form of increased thermal dissipation of photoexcited chlorophyll, which displays a major component of NPQ, occurs within tens of seconds to minutes (4), while plant tropism takes longer time scales of hours to days (3, 5). Another photoadaptation mechanism, at a single-cell level, includes the motion and rearrangement of chloroplasts to optimize light absorption or to avoid photodamage (2, 6–10).

Such strategies are fundamental for survival in fluctuating environments, displaying interesting features such as computation and integration in the context of plant phototropism (11–13), harnessing noise for optimal shade avoidance (14), and dynamical phase transitions of chloroplast motion exhibiting features of active glasses (10). In land plants, the collective motion of individually sensing and moving disk-shaped chloroplasts (9, 15) leads to large-scale rearrangements inside leaf cells, aiming to optimize light uptake while avoiding strong light. However, a vast amount of photosynthesis happens in aquatic environments, especially the oceans (16–18). Some single- and multicellular algae and cyanobacteria can actively move collectively or individually as a response to light (19–28). In contrast, nonmotile photosynthetic organisms have evolved various different strategies for photoadaptation, such as repositioning their chloroplasts, similar to plants, within their cell bodies (29, 30).

Here, we study the light adaptation strategy of the nonmotile marine dinoflagellate *Pyrocystis lunula*. Dinoflagellates are a large and diverse group of photosynthetic and mixotrophic algae (31, 32) that underwent tertiary endosymbiosis by engulfing algae containing a secondary plastid (33, 34). This process led to the formation of chloroplasts with three or more membranes (34, 35).

*Pyrocystis* spp., a group of bioluminescent dinoflagellates, inhabit warm oceanic and coastal waters worldwide, with high abundance in the euphotic zone at depths between 60 and 100m (36, 37). Living in such depths means that photosynthetically available light is reduced to as little as 1% of surface light. The approximately 100μm-sized crescent-shaped dinoflagellate *P. lunula* spends most of its life as a nonmotile cyst (38, 39). During the night, *P. lunula* exhibits mechanically induced bioluminescence (40–42), which serves as a defense mechanism (43). To achieve this, *P. lunula* reorganizes its internal architecture, switching from a photosynthetic day phase to a bioluminescent night phase, facilitated by the active movement of chloroplasts and bioluminescent organelles (scintillons) toward and away from the cell center in accordance with a circadian rhythm (39, 44, 45).

Interestingly, the same intracellular rearrangement can be triggered during the day-phase by strong light (46), leading to a rapid compression of the chloroplast within a few minutes, without any deformation of the thick cell wall (38).

How is such a rapid and drastic intracellular rearrangement orchestrated within the confinement of the cell wall? Here, we uncover the dynamics of chloroplast motion as a light adaptation mechanism in *P. lunula*. We unravel its active reticulated chloroplast structure, enabling *P. lunula* of fast intracellular morphological reorganization, by facilitating the organelle to undergo significant deformations within the confinement of the cell wall. The topologically complex structure of chloroplasts is, therefore, crucial for efficient photoadaptation. We find that the dynamic features of this photoavoidance motion in response to environmental changes are, in their core, similar to the photoadaptation motion of chloroplasts in plants, despite the distinct origins of chloroplasts in dinoflagellates and plants (33, 34, 47).

## Chloroplast Contracts under Strong Light

**Fig. 1.**
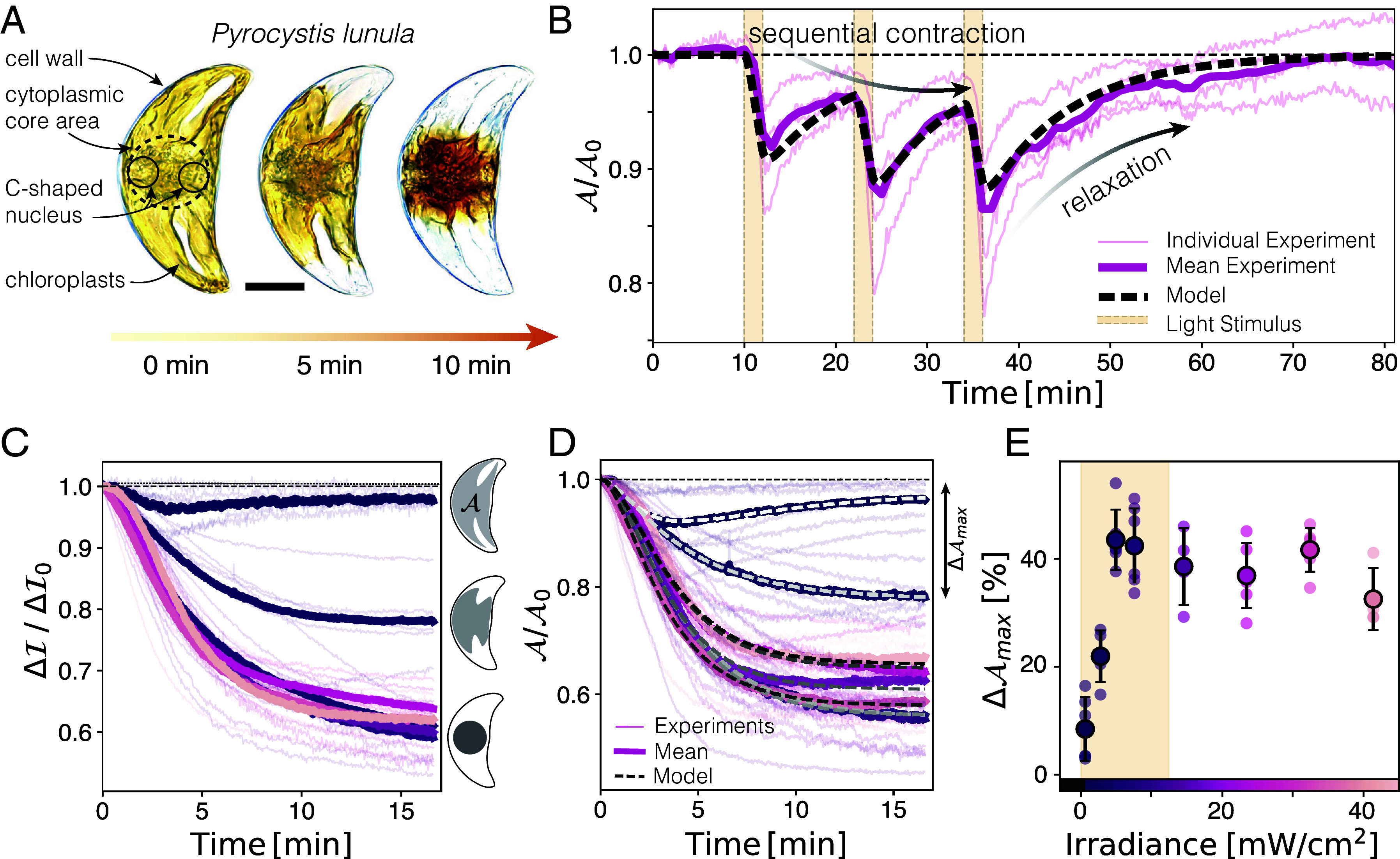
Chloroplast area undergoes rapid changes under strong light illumination. (*A*) Upon light activation, the chloroplast (yellow) contracts and compacts (darker color) in less than 10min. (Scale bar: 20μm.) (*B*) Periodic white light stimulation (yellow bars), causes the chloroplast area A (normalized by initial area $A_{0}$, N=4, pink curve: mean) to decrease under strong light and expand under dim light. The dynamics are well described by an active viscoelastic model (dashed line). (*C* and *D*) Relative light loss and area decline in response to varying light intensities in specimens initially adapted to dim light, indicating increased light transmission in the light-avoidant state. Higher light intensities cause a stronger response (colorscale in *E*). (*E*) The maximum area reduction correlates (Pearson’s ρ=0.897) with ecologically relevant light irradiance ($I<12\;\;{\text{mW/cm}}^{2}$, yellow box). Beyond ecologically relevant light conditions, the contraction response saturates to a maximal decrease of the chloroplast area by 40%.

We studied the adaptation of the chloroplast area of individual *P. lunula* cells to white-light exposure under physiologically relevant irradiances of its natural habitat at a depth of 60to100m (37). The spectrum of white-light used compares to oceanic underwater light conditions in the euphotic zone (*SI Appendix*, Text I and Fig. S1) (48, 49), especially with a strong peak in the blue light regime. Previous studies showed that the growth of *P. lunula* reaches full saturation under low light conditions found at approximately at 50m depth, equivalent to about $12\;{\text{mW/cm}}^{2}$ (36), suggesting optimal light conditions below this value. We study the cell response to white-light stimulation from 2.8 to $41.4\;{\text{mW/cm}}^{2}$, and show chloroplasts move toward the cytoplasmic core area (photoavoidance) within approximately 10min (Fig. 1*A* and *SI Appendix*, Fig. S1), in line with earlier observations (46).

We measure the projected area of chloroplasts, denoted as A(t), and the light transmission through the cell, I(t), over time. To quantify dynamic changes in the absorption and reflection, we calculate a loss value: the difference of transmitted light and incident light estimated by the background intensity $I_{\mathrm{bg}}$ relative to the initial loss: $\frac{I_{\mathrm{bg}}-I\left(t\right)}{I_{\mathrm{bg}}-I\left(0\right)}=\Delta I/\Delta I_{0}$ (*Materials and Methods*). This value provides a relative measure of the increase of transmitted light over time.

During the light-adaptation motion, the relative loss (Fig. 1*C*) and the projected area A of the chloroplast (Fig. 1*D*) exhibit similar dynamics for various light irradiances (*SI Appendix*, Text II and Fig. S2 and Movie S1). After a short time of approximately 1 to 2 min, characterized by a positive curvature, both the projected area and the relative loss follow an exponential decay, eventually reaching saturation at levels dependent on the light intensity. These measurements are related: As the chloroplast retracts, more light can pass through the cell, leading to reduced loss by up to 40%. This decrease is linked to a reduced absorption, which leads to less potential photodamage and is, therefore, of high physiological relevance for the organism. Contrary to the contraction scenario described above, exposure to dim white light ($0.6\;\;{\text{mW/cm}}^{2}$) triggers a transient response wherein the chloroplast initially contracts toward the cell center but eventually expands again (Fig. 1*D* and *SI Appendix*, Fig. S2*A*). These observations share similarities with terrestrial plants’ transient photoresponse at intermediate light intensities (50, 51).

Stimulation irradiances within the ecologically relevant light conditions of 0.2 to 12 $\;{\text{mW/cm}}^{2}$ (37, 46) and the culturing conditions $I=0.27\;\;{\text{mW/cm}}^{2}$ show a gradual decrease of the chloroplasts projected area (Pearson’s ρ=0.897), suggesting that the chloroplast contraction response evolved as an adaptive trait. Strong irradiances ($I>12\;\;{\text{mW/cm}}^{2}$) lead to the saturation of the maximal contraction $\Delta A_{\;\text{max}}\approx 40$%, restraint by the size of the cytoplasmic core area (Fig. 1 *A* and *E* and *SI Appendix*, Fig. S2*C*). Irrespective of the applied light levels, the dynamics of area reduction have a similar shape. In the following, we extract the relevant time scales of these intercellular rearrangements by probing the organism’s response to dynamic light variations.

## Dynamic Testing and Modeling of Chloroplast Contraction

Alternating strong white- and dim red-light irradiation controls the chloroplast contraction and relaxation dynamics toward and away from the cytoplasmic core area (Fig. 1*B* and *SI Appendix*, Fig S4 *A*–*D* and Movies S2 and S3): The chloroplast network rapidly responds to white-light (photoavoidance) and slowly relaxes upon red-light exposure (photoaccumulation). Long-time illumination with weak red light ($0.2\;\;{\text{mW/cm}}^{2}$) leads to a complete expansion of the chloroplasts over the entire cell (Fig. 1*B*, Time >50min, and Movies S2 and S3).

We develop a mathematical model (*SI Appendix*, Text III and Fig. S3) to fit our dynamical tests (dotted lines Figs. 1 *B* and *D*, 3*G*, and 4 *C* and *D* and *SI Appendix*, Fig. S4). Specifically, we model the relative projected chloroplast area $A\left(t\right)/A_{0}=1-x\left(t\right)$, where x(t) corresponds to a relative extension of the chloroplast (x=0 if the chloroplast is fully extended). In our experiments, we observe a dynamic (noninstantaneous) contraction to the onset of light (Fig. 1*D*), which is modeled as a Kelvin-Voigt viscoelastic material with response time scale $\tau_{\mathrm{KV}}$

[1] $$\begin{array}{c} \tau_{\mathrm{KV}}\frac{\;\text{d}x}{\;\text{dt}}+x=p\left(t\right). \end{array}$$

The light-induced contractile forcing will move the chloroplast to a new equilibrium position p(t) within a timescale $\tau_{\mathrm{KV}}$. Notably, we found different timescales: $\tau_{\mathrm{KV}}$ for contraction ($\frac{\;\text{d}x}{\;\text{dt}}>0$) and $\tau_{\mathrm{KV}}^{*}$ for expansion ($\frac{\;\text{d}x}{\;\text{dt}}<0$). The enforced position p(t) is modeled by a light-sensory process with two light sensors, of which one sends signals $c_{1}\left(t\right)$ at all light intensities, while the other sends chemical signals $c_{2}\left(t\right)$ at intensities below a threshold $I_{\mathrm{th}}$. The generation of the two chemical signals within time scales $\tau_{1}$ and $\tau_{2}$ is modeled by first-order chemical processes:

[2] $$\begin{array}{c} \tau_{1,2}\frac{\;\text{d}c_{1,2}}{\;\text{dt}}+c_{1,2}=s_{1,2}\left(I\left(t\right)\right). \end{array}$$

Here $s_{1,2}\left(I\right)$ are two photosensory functions of the time-dependent light stimulation I(t). The first sensor sends a light-avoidance-signal $c_{1}$ inducing the contractile force acting on the chloroplast material (here, we assume $s_{1}\left(I\left(t\right)\right)=\alpha_{1}I\left(t\right)$, where $\alpha_{1}>0$ is a constant). The second sensor generates a repressive signal $c_{2}$ which opposes the contractile signal $c_{1}$ and can account for the transient response (Fig. 1*D*). This is similar to transient responses by the integration of opposing signals from phototropin 1 and 2 in *Arabidopsis thaliana* (50) (*SI Appendix*, Text III). We observe the transient response only at the smallest irradiance setting ($I=0.6\;\;{\text{mW/cm}}^{2}$, Fig. 1*D* and *SI Appendix*, Fig S2*A*), hence we require that the second light sensor only signals at low light intensities, which we describe with the light-response function $s_{2}\left(I\right)=\alpha_{2}H\left(I_{\text{th}}-I\right)$, where H is the Heaviside step function, i.e., $H(I\geq I_{\text{th}})=0$ and $H(I<I_{\text{th}})=1$ and $\alpha_{2}>0$ is a constant. The threshold intensity lies between $I_{\text{th}}=0.6$ and $2.8\;\;{\text{mW/cm}}^{2}$. Taken together, the two signals will lead to the generation of a force to move the chloroplast material to a new equilibrium position $p\left(t\right)=\beta (c_{1}\left(t\right)-c_{2}\left(t\right))$ (β>0). We solve this model analytical for constant light in the case of low light ($I<I_{\text{th}}$) and strong light ($I\geq I_{\text{th}}$) (*SI Appendix*, Text I) and find that the chemical signaling of a photoavoidance response occurs at a time scale of $\tau_{1}=1.74\;\pm 0.8\;\;\text{min}$ (mean ± SD, N=42), while the repressing signal leading to a transient response occurs at a similar time scale of $\tau_{2}=1.76\;\pm 0.3\;\;\text{min}$ (N=6). The timescales $\tau_{\mathrm{KV}}$ and $\tau_{\mathrm{KV}}^{*}$ emerge from the dynamics of the driving mechanism, relying on actin, microtubules, and molecular motors such as myosin (45) (also see *SI Appendix*, Text IV and Fig. S5 for inhibitory treatment), and are found to be $\tau_{\mathrm{KV}}=2.25\pm 0.8\;\;\text{min}$ (N=42) for contraction and $\tau_{\mathrm{KV}}^{*}=7.2\pm 0.7\;\;\text{min}$ (N=6) for expansion of the chloroplast. Hence, the cell undergoes a rapid photoavoidance to strong light and a slow photoaccumulation response toward dim light (*SI Appendix*, Fig. S4 and Tables S1 and S2).

Notably, our model can be interpreted as a low-pass filter for environmental stimuli (*SI Appendix*, Text III and Fig. S3*E*), analog to a Butterworth filter (52). High-frequency noise, exceeding a frequency of about $\omega \approx 0.3\;\;{\text{min}}^{-1}$ does not trigger a strong response (*SI Appendix*, Figs. S3*E* and S4*D*), while long-time stimulation gives the organism enough time to adapt (*SI Appendix*, Fig. S4*C*). Hence, this system has likely evolved to effectively filter out potentially irrelevant high-frequency light fluctuations like surface waves [ω≈0.1to1Hz (53)], while being adaptive toward changes in light by the movement of clouds (54, 55) or circadian vertical migration (56). Additionally, the difference of the time scales $\tau_{\mathrm{KV}}$ and $\tau_{\mathrm{KV}}^{*}$ allows the organism to rapidly respond to potentially harmful light conditions, but to slowly adjust the chloroplast area in low light.

While the model successfully recovers *P. lunula*’s overall light response, it cannot resolve the underlying mechanical features leading to such a drastic deformation of the chloroplast within the confinement of the stiff cell wall. To understand the dynamics of chloroplast motion, we need to unravel the structural properties that facilitate such intracellular rearrangements.

## Topologically Complex Chloroplast Network Allows Efficient Contraction

The contraction of the projected area reaches up to 40% of the cell area, necessitating a remarkably large deformation of the cytoplasmic material. Such deformations within hard confinement, in this case, constituted by the cell wall of *P. lunula* (38), are subject to physical constraints. To elucidate these constraints, we draw analogies to the compression of fluids and solids: An incompressible fluid cannot “contract” uniformly toward the center under confinement, as its volume must be conserved. Similarly, an elastic solid, when compressed from one direction, will expand in orthogonal directions, a behavior characterized by a positive Poisson’s ratio, comparing the strain perpendicular to the direction of loading to parallel strain. Isotropic incompressible elastic materials in three dimensions have a Poisson’s ratio of ν=0.5 (57). Under confinement, this property (ν>0) leads to effective strain-stiffening, as the expansion into orthogonal directions is prevented. However, structured metamaterials defy positive Poisson’s ratios by allowing nonlinear deformations such as buckling or the action of rotating hinges (58–60). Auxetic material behavior, characterized by ν<0, can be found in polymer networks (61), foams (62) and engineered meta-materials. Such materials, including poro-elastic materials such as cork (ν≈0) (63), enable uni- or multidirectional contraction, facilitating efficient deformations even within confined spaces. Below, we will show evidence that cytoplasmic space of *P. lunula* containing the chloroplasts exhibits such metamaterial-properties.

**Fig. 2.**
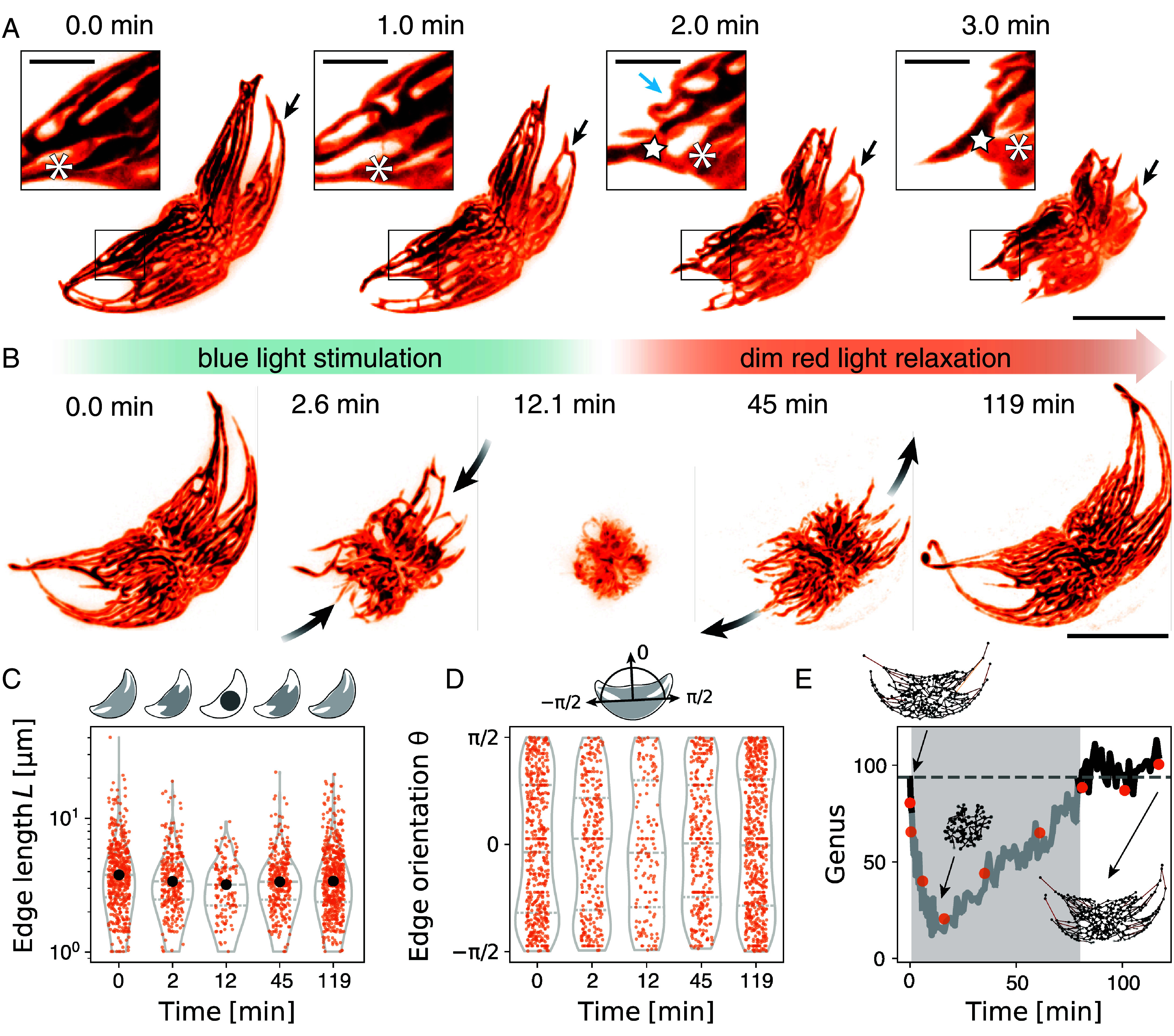
Dynamic light-controlled contraction and expansion of the chloroplast reticulum. Autofluorescence imaging of chloroplasts reveals reticulated structure. (*A*) Light stimulation beginning at t=0min causes the chloroplast network to contract by moving strands toward the cells’ center and deforming them (arrows). A compact structure is achieved by reducing the hole sizes of the network. (Scale bar: 40μm.) *Inset*: Examining an area of dynamical deformation of a strand of the chloroplast network, two nodes (asterisk and star) change their position as well as their distance from one another. The blue arrow indicates a deforming cytoplasmic strand leading to the closure of a hole in the network. (Scale bar: 10μm.) (*B*) Chloroplast contraction under blue light stimulation and subsequent expansion under the dim red light environment. (*C*) Edge length statistics of the chloroplast network (from *B*) over time. Large edges shorten and extend again. (*D*) Distribution of orientation of edges. Orientations align with the major axis of the cell, but during contraction, the distribution flattens. (*E*) Topological measurements over time indicate the similarity of structures before and after the stimulation. Black line: Genus estimated by Betti number $\beta_{1}$ of the network. Orange points: Topological genus measured from Euler characteristic χ of the mask image. During contraction, chloroplast strands touch and seemingly close holes, leading to an apparent decrease in the detected genus (gray area). Genus before contraction and after expansion have similar values.

Using confocal autofluorescence imaging of the chloroplasts (*Materials and Methods*) we uncover a chloroplast structure is well described as a reticulum (latin: small network) (Fig. 2*A*). This intricate network shows similarities to early observations made in the context of spontaneous diurnal chloroplast relocations (39, 45) and recently observed rapid changes of buoyancy in the related species *Pyrocystis noctiluca* (56). Continuous blue light stimulation (λ= 470 ± 50 nm) of the cell, induces contraction of the chloroplast network over time. We uncover two mechanisms which choreograph this chloroplast photoadaptation motion: Cytoplasmic strands move toward the center while they simultaneously contract in a manner reminiscent of buckling (Fig. 2*A* and Movies S4–S6), enabling a unidirectional contraction of the entire structure. Buckling, or inward-folding, allows the structure to compact into the space between the cytoplasmic strands (ν≲0), circumventing strain-stiffening typically expected from uniform bulk contractions under confinement if ν>0. Moreover, we observe a notable thickening of some strands during contraction, indicating a flow of material within the structure (Fig. 2*A* and Movies S4–S6).

**Fig. 3.**
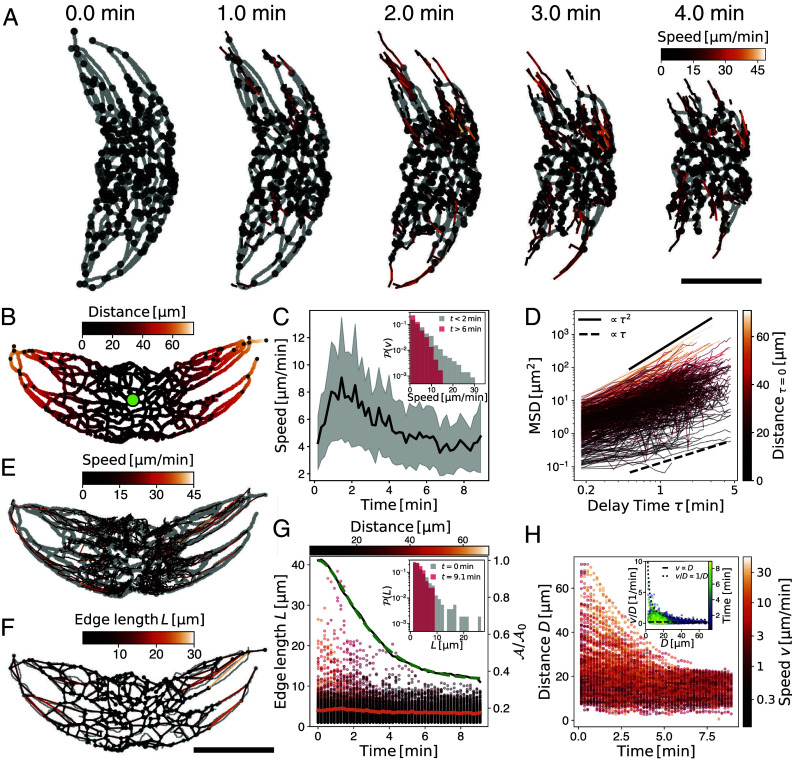
Network dynamics of the chloroplast reticulum. (*A*) Time series of chloroplast contraction dynamics. The nodes (black dots) of the underlying three-dimensional network move on paths inward (color: speed). Gray: 2d projection of the underlying skeleton. (*B*) Distance-map from the center point (green dot) on a 2d-projected mask of the experiment, as in (*A*) at t=0min. (*C*) Average individual node speed (black line: mean ± SD) increases during contraction and subsequently decreases. *Inset*: The speed distribution is heavy-tailed. Initially fast trajectories (t<2min) slow down at long times t>6min. (*D*) Mean-squared displacement (MSD) of individual trajectories ranges from diffusive trajectories (MSD∝τ) in the center (colors corresponding to the distance from the center (*B*) at the beginning of the trajectory) to purely ballistic trajectories ($\;\text{MSD}\propto \tau^{2}$) originating at the periphery. (*E*) Trajectories are mapped on the skeleton of the mask (gray) and colored according to their local speed, reaching up to 30μm/min. (*F*) Network representation of the chloroplast. Edges are colored by length. The network closely represents the skeleton of the mask (gray). (*G*) The distribution of edge lengths of the network decays over time. The mean edge length (orange line) is hardly affected. Colorbar: edge distance to the center corresponding colors in (*B* and *D*). The shrinkage of the longest edges follows a similar trend to the decrease in the relative projected area of the chloroplast network (black line, *Right* axis). The model fits to the projected area curve (dashed green line) and is in agreement with the experimental observations. *Inset*: The network has initially (t=0min) a few very long trajectories (gray), which disappear at long times (red). (*H*) Correlation of distance and speed. Far-distanced nodes travel at higher speeds (brighter colors) than centrally located nodes. Note the logarithmic colorbar. *Inset*: “Strain rate” (speed over distance) of every node is constant at large distances. The dashed line corresponds to v∝D, which is expected for elastic deformation at a constant strain rate. The dotted line corresponds to constant v. Colormap represents time. (Scale bars: 40μm).

Under dim red light, the chloroplast expands again over a longer time scale (Fig. 2*B* and Movie S6). Although the chloroplast network appears slightly different before and after the contraction and expansion, the main geometrical features remain unchanged: When fully spread, the network is characterized by a few very long cytoplasmic strands (Fig. 2*C*), which extend outward along the cell’s long body axis (Fig. 2*D*). During contraction, these distinctive features are lost as the chloroplast reticulum obtains a more spherical shape. Remarkably, despite these morphological changes, the network topology remains largely unchanged before contraction and after expansion, suggesting a permanent connection among nodes without dynamic rewiring of the network (Fig. 2*E*). The apparent loss of holes, as indicated by the decline in topological genus, can be attributed to the increased contact between strands, which complicates the identification of individual strands. We segment and skeletonize the microscopic images at all times and extract a network representation (edges and nodes) of the chloroplast. This allows us to analyze the spatiotemporal dynamics of the networks’ nodes over time (Fig. 3*A*, *B*, and *E* and *Materials and Methods* and Movie S7) and to quantify the contraction process in detail. Our findings reveal a wide distribution in node speeds, which vary over time (Fig. 3 *A* and *C*). Predominantly, nodes close to the cytoplasmic core area exhibit slow, diffusive motion characterized by a time-linear mean-squared displacement (MSD∝τ). Conversely, nodes located farther from the cell center move ballistically ($\;\text{MSD}\propto \tau^{2}$) (Fig. 3*D*). Thus, longer strands, initially located far from the cytoplasmic core area, move rapidly toward the center (Fig. 3 *E* and *H*), while reducing their length, resulting in the reduction of the chloroplast’s 2D-projected area A (Fig. 3*G*). Interestingly, the strands follow along their initial configuration, as indicated by the overlay of trajectories on the network’s 2D projection at t=0min in Fig. 3 *A* and *E*. This may reflect a signature of their actin- and microtubule-mediated driving mechanism (45) and a coalignment to those networks.

To elucidate whether actin and microtubules play a significant role during the light-mediated chloroplast contraction, we treat dark and light-adapted cells with the actin depolimerizer Latrunculin B, Nocodazole (microtubule depolimerization) and 2,3-Butanedione monoxime (myosin-II inhibition) for their response upon strong white light and dim red light for their photoadaptation response. We found that actin is essential for the motion (*SI Appendix*, Fig. S5), while the role of microtubules and myosin remains unclear (*SI Appendix*, Text IV).

Further analysis of the network, demonstrates that the speed of the nodes scales with their distance from the center, v∝D, aligning with the expected behavior from the one-dimensional contraction of an elastic material under a constant strain rate (Fig. 3*H*, *Inset*). This observation confirms that the complex topology of the network enables the material to contract, dominantly, along one dimension. These findings have been valuable in developing the mathematical model (*SI Appendix*, Text III).

### Chloroplast contraction shows local and global responses to local stimulation.

To further shine light on the sensing mechanism, we locally stimulate the cell (*Materials and Methods*) with a blue low-power laser (λ=488nm, $3.25\;\;{\text{mW/cm}}^{2}$). This wavelength closely matches the optimal absorption peak of phototropins (64), which are crucial photosensors for chloroplast positioning in terrestrial plants (9, 65, 66) and also present in *P. lunula* (67), potentially accounting for the observed strong response.

**Fig. 4.**
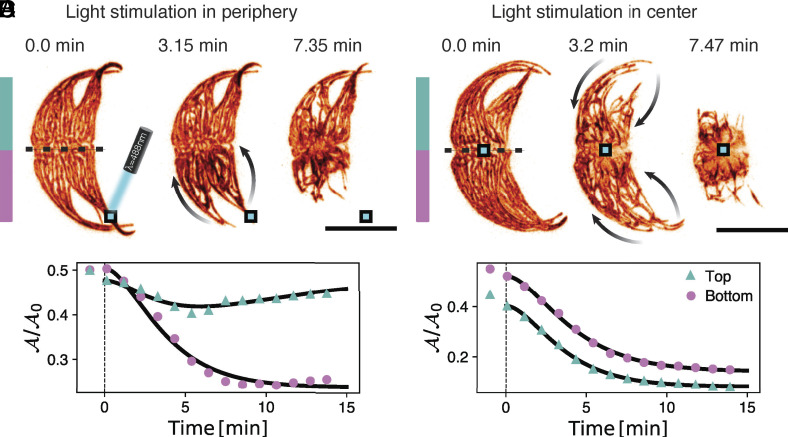
Local sensing leads to local or global response. (*A*) Local stimulation in the periphery with blue light (λ=488nm, $I=3.25\;\;{\text{mW/cm}}^{2}$) leads to a one-sided response away from the stimulation region within a few minutes. Note the contraction of the network in the opposite site. The blue square indicates the stimulation region. (*B*) Light stimulus in the center (blue square) leads to the global movement of the chloroplast network toward the center (arrows). (*C*) Area contraction in (*A*) *Upper* and *Lower* half of the cell (divided by the dashed line in *A*). The *Lower* part (at the stimulus site) contracts rapidly, while the *Upper* half undergoes a transient response. (*D*) Area contraction in (B). The *Upper* and *Lower* half of the cell undergoes a symmetric response. Black lines: contraction model. See Movies S8–S10. (Scale bar: 40μm).

We illuminate a $6.8\times 6.8\;\mu \;{\text{m}}^{2}$ region either in the center or periphery of the algae, respectively (Fig. 4 *A* and *B* and Movies S8–S10). Peripheral stimulation in one side of the chloroplast network leads to a localized contraction of this side toward the cytoplasmic core area (Fig. 4*A*). The opposite side of the chloroplast network moves less pronounced, but within the temporal resolution limit of 20to40s. This rapid onset suggests that a fast diffusing signal triggers a long-range response across the cell of length L≈80to100μm within a time of τ≈20to40s after light-stimulation begins, implying a diffusion coefficient on the order of $D\approx L^{2}/\tau \approx 160\;\;\text{to}\;500\;\mu \;{\text{m}}^{2}/\;\text{s}$. Interestingly, the chloroplast retraction on the nonstimulated region is transient or less pronounced, as observed in small light intensity stimulation (Fig. 1*D* and Movies S8 and S9), suggesting that the transmitted signal is depleted, as expected for diffusive signaling.

Surprisingly, upon central stimulation in the cytoplasmic core area, all chloroplast strands move toward the cell center, within similar time scales (Figs. 1 *B* and *D* and 3 *A* and *G*). This suggests, that irrespective of the location of the light stimulus, the chloroplast network moves toward the center: during peripheral stimulation away from the stimulus side and during central stimulation toward it.

These findings suggest, that a nonspecific signaling molecule spreads rapidly throughout the cell to trigger the contraction mechanism.

## Discussion

In our experiments, we show that the chloroplasts of *P. lunula* retract toward the cell’s center under strong white or blue light conditions and expand under weak red light conditions. This bidirectional movement of chloroplasts toward and away from light, mirrors the chloroplast photorelocation motion seen in leaves of green plants (2, 6, 9, 50), nonetheless, using a fundamentally different mechanism. The significant light-induced retraction of chloroplasts leads to increased light transmission through the cell (Fig. 1*C*), suggesting that, similar to green plants, *P. lunula* employs this mechanism as a means of light avoidance (2, 6, 7, 9). Notably, under low white light conditions, we observe a transient response in the chloroplasts—initial fast contraction followed by slow expansion—suggesting a dynamic competition between these processes. This shows similarities with the observed counteraction of phototropin 1 and 2-mediated chloroplast motion (50, 68). In fact, the transcriptome of *P. lunula* bears various phototropin 1 and phototropin 2-like sequences and Light-oxygen-voltage-sensing (LOV) domains (67), pointing toward potential similarities in light sensation. The similarity of this organism’s light response to the one of green plants is surprising, as the origin of chloroplasts in dinoflagellates is very distinct: While green plants obtained their chloroplasts from primary endosymbiosis of cyanobacteria, dinoflagellates underwent tertiary endosymbiosis, including the incorporation of a red-algae (33, 34, 47).

At light intensities exceeding the natural physiological conditions of dinoflagellates, chloroplasts contract fully toward the cell center. Under such extreme conditions, the crowding of the chloroplast strands poses a mechanical limit for contraction, and consequently for this photoavoidance mechanism. However, within their native physiological light conditions, *P. lunula* responds via light-dependent chloroplast compaction, indicating the evolution of a gradual adaptation response to various ecologically relevant light conditions. Furthermore, we find that the relaxation phase following strong light-induced contraction occurs over a longer time scale, suggesting a different driving mechanism for the expansion of the chloroplasts.

The large-scale transport of organelles observed likely depends on the coordinated action of the actin and microtubule networks, together with molecular motors such as myosin, as has been demonstrated in the context of the diurnal intracellular reorganization between the photosynthetic phase during the day and bioluminescent phase at night (45), in line with pharmacological perturbation experiments for light-adaptation (*SI Appendix*, Text IV and Fig. S5) in which we confirm that actin is necessary for bidirectional chloroplast motion. However, the exact driving mechanism of the chloroplast relocation in *P. lunula* remains unidentified and may differ from that of green plants, where chloroplast movement is primarily driven by the assembly of short actin (cp-actin) filaments and the transmission of polymerization forces toward the plasma membrane (9, 15, 68).

We elucidate the details of the reticulated morphology of chloroplasts and show that such a structural “design” offers mechanical advantages. Structured metamaterials, like this chloroplast morphology, facilitate buckling and other complex deformations (58, 59, 69), enabling efficient chloroplast contraction under the confinement of the cell wall. However, to strengthen this analogy, a more in-depth study of the organelles’ architecture and contractile apparatus is required. Interestingly, *P. noctiluca*, another species within the *Pyrocystis* genus, was found to have a reticulated cytoplasm, assisting with the vertical migration (56), showing another intricate link between the morphology of cytoplasmic space and its function in dinoflagellates.

We also showed that the dynamics of chloroplast contraction can be effectively modeled using a “viscoelastic” framework with chemically controlled stress applications. Although our coarse-grained model does not pinpoint the precise origins of observed elasticity and viscosity—whether from passive or active cellular components—it importantly enabled us to identify adaptation time scales and compare them with the ecologically relevant fluctuations. These time scales suggest that chloroplast motion serves as a feasible light adaptation strategy for environmental light variations persisting longer than 3to5min. Indeed, such fluctuations notably exceed the duration of second-long light changes induced by waves but are in line with the motion of clouds obscuring the sun (53) and might complement nonphotochemical quenching.

Our experiments have also shown that chloroplast contraction is driven by local sensing, with directed relocation observed when chloroplasts are locally stimulated (Fig. 4). However, even though locally stimulated, the chloroplast network on the opposite side of the cell contract within 20to40s, indicating a long-ranged signal transfer via fast diffusive signals ($D\approx 160\;\;\text{to}\;500\;\mu {\text{m}}^{2}/\;\text{s}$), such as calcium, recognized for its important roles in photosensory downstream signaling (66) and in *P. lunula* bioluminescence (70). Interestingly, stimulation in the cytoplasmic core prompts chloroplasts to move toward rather than away from the center. This counterintuitive behavior might result from the network’s topologically conserved structure and a photo movement that is inherently biased toward the cell center. This hypothesis, however, needs further investigation. Moreover, the intricate relationship between the chloroplast and nucleus, with the latter hosting a significant portion of the chloroplast genome (2, 71), suggests that chloroplast movement toward the nucleus in the cytoplasmic core (39) could also serve a photoprotective function, shielding genetic material from intense light damage. Our study provides evidence for such a mechanism in dinoflagellates, however, a comprehensive examination of the chloroplast-nucleus relationship is necessary to fully understand these dynamics.

Overall, the complex relationship between the geometry and topology of chloroplast structure and its dynamics provides a fertile ground for exploring intriguing physical dynamics with significant physiological implications, in the context of light–life interactions.

## Materials and Methods

A detailed description of the mathematical model and fitting procedures, as well as the pharmacological treatments is given in the (*SI Appendix*, Text). An overview over data analysis and experimental procedures is given below.

### Cell Culture.

*P. lunula* (Schütt) is cultured in f/2 medium in an incubator (Memmert) at $20^{\;{}^{\circ}}\;\text{C}$ and a 12:12 day-night cycle with light irradiance at $0.27\;\;{\text{mW/cm}}^{2}$.

### Brightfield Microscopy.

We perform bright-field microscopy with a Nikon TI2 E microscope. Images are acquired using a Photometrics BSI Express camera at a frame rate of 1to2frame/s. We use a 40×/1.2 NA PLAN Apochromat objective and simultaneously measure 3 to 5 different positions using a motorized xy-stage totaling 4 to 8 cells for every light intensity setting ($0.6,2.8,4.9,7.6,14.5,23.4,32.4,\;\;\text{and}\;41.4\;\;{\text{mW/cm}}^{2}$). To allow for chloroplast expansion, imaging was performed using a red filter with a cut-on wavelength of $\lambda_{c}=625\;\;\text{nm}$, at light intensities of $0.1\;\;\text{to}\;0.4\;\;{\text{mW/cm}}^{2}$. Periodic white ($7.6\;\;{\text{mW/cm}}^{2}$) and red light ($0.1\;\;{\text{mW/cm}}^{2}$) stimulation was controlled through a custom script in microManager (72).

### Confocal Microscopy.

Global simulation experiments are performed with a Nikon Ti Eclipse microscope equipped with a 60×/1.49 NA or 40×/1.3 NA oil objective, a confocal spinning disk unit (Yokogawa CSU-X1) with a microlens array, and an Andor iXonEM+ 897 electron-multiplying charged-coupled device camera. Chlorophyll autofluorescence is stimulated at 640nm wavelength while the emission bandpass filter (680to740nm) is used. By using a piezo z-stage we record 50to120 z-steps (step size 0.3to1μm) within 11to20s with a 30to60ms excitation time. The brightfield path of the microscope was equipped with a 470±50nm blue filter to stimulate the cell at an intensity of $0.6\;\;{\text{mW/cm}}^{2}$.

### Local Stimulation.

Local stimulation experiments are performed using a 63× PLAN APO IR objective on a Nikon TI body with a Nikon A1 resonant scanner. Imaging is performed using a 637nm laser line and emission filter at 650nm. A whole z-stack consists of 80 to 100 steps of 0.5μm size. The pinhole diameter is fixed to 1.2 Airy disk diameters. Local stimulation is performed by applying a 488nm laser at low irradiance $3.25\;\;{\text{mW/cm}}^{2}$ in a small scanning region of $6.8\times 6.8\;\mu {\text{m}}^{2}$. One z-stack takes between 45s and 60s and the stimulation time is 3.9s for center-stimuli or 7.8s for peripheral stimuli.

### Image Processing.

Image analysis was performed in Fiji (73) and Python using Napari (74) and scikit-image (75). In the following paragraphs, we will outline the different image-processing steps for the data we acquired.

#### Measuring chloroplast area and transmittance from brightfield data.

We customized a Fiji macro, which measures brightfield intensity I in a hand-annotated region of interest (ROI) for sequential stimulation experiments, or generated ROI for constant illumination experiments. The ROI is generated by Gaussian smoothing of the first frame with a 21 px kernel and subsequent thresholding using the triangle method. Morphological closure with a 51 px-diameter disk-shaped mask helps filling holes within the mask. To measure the area, we calculate the mean value of the background, subtract it by one SD and employ it as a threshold value to discriminate the chloroplast material from the background light transmitted through the organism. To measure the loss of light (absorption, reflection) we calculate $\Delta I\left(t\right)=I_{0}-I\left(t\right)$, where the incident light $I_{0}$ is estimated from a background intensity $I_{0}\approx I_{\mathrm{bg}}$ measured within a ROI outside of the cell. To normalize we calculate the relative of loss compared to the initial loss at time t=0min: ΔI(t)/ΔI(0).

#### Analyzing 3D confocal data.

We segment and skeletonize 4d stacks (x,y,z,t) by using a script developed within Napari. The following steps are performed for all time steps. First, we estimate the loss of light intensity deeper within the sample by fitting the Beer–Lambert law $I\left(z\right)=I_{0}e^{-z/\lambda }$ and correcting the z-stack accordingly. Next, we blur the x-y plane with a 1 px-wide Gaussian and subsequently use a top-hat filter with a (2.8,2.8,1)μm kernel. Then, we use the triangle method to threshold and perform binary closing with a (1,1,1)μm kernel. We label the obtained mask with a connected-component method and reject small labels <20,000 px. Three-dimensional graph analysis was performed after skeletonizing the label image (76) and generating a NetworkX graph (77). Some closely located nodes of the graph are merging over time, hence we coarse-grain nodes that are less than 5 px away from each other by deleting them and setting one node in the middle between them. Similarly, we reject end-nodes that have a single edge less than 9 px long. The nodes of the graph represent the junctions of the chloroplast reticulum and are tracked using a nearest-velocity tracker in trackpy (78) with a (6μm,6μm,and2μm) search window and 4-time step memory (≈40 to 60 s), as well as an adaptive search window to down to 10% of the initial search window size, to enhance the trajectory matching in a dense environment. Trajectories with less than 9 timesteps, corresponding to about 90to120s, are rejected. We calculate velocities using a Savitzky–Golay filter using a 30 to 45 s window to fit second-order polynomials and take a smooth derivative. The MSD of individual trajectories is calculated by a time average $\;\text{MSD}\left(\tau \right)={\left\langle \vec{x}\left(t+\tau \right)\vec{x}\left(t\right)\right\rangle }_{t}$ up to a maximal displacement of half the trajectories length (τ≤T/2). We calculate the edge lengths between every pair of nodes on the graph. Betti numbers $\beta_{1}$ calculated on the graph and are related to the topological genus by $g=\beta_{1}/2$. The Euler characteristics χ are calculated from the mask using region properties of scikit-image (75) and relate to the topological genus g=1−χ/2.

## Supplementary Material

Appendix 01 (PDF)

Movie S1. Chloroplast contraction of *P. lunula* at different light intensities: (left) 0.6mW/cm^2^, (center) 2.8mW/cm^2^ and (right) 41.4mW/cm^2^, corresponding to S2A-C, respectively.

Movie S2. Dynamic light-controlled chloroplast motion with 10min 0.4mW/cm^2^ red-light imaging between 2.5min-lasting white-light stimulation at *I* = 7.6mW/cm^2^.

Movie S3. Dynamic light-controlled chloroplast motion with 15min 0.4mW/cm^2^ red-light imaging between 15min-lasting white-light stimulation at *I* = 7.6mW/cm^2^.

Movie S4. Time series for global stimulation with blue light (470 ± 50 nm. Imaging of chlorophyll auto-fluorescence (red look-up table (LUT)).

Movie S5. Time series for a second global stimulation with blue light (470 ± 50 nm. Imaging of chlorophyll auto-fluorescence (red look-up table (LUT)). The sequential buckling of cytoplasmic strands is clearly visible.

Movie S6. Time series for a global stimulation with blue light (470 ± 50 nm. Imaging of chlorophyll auto-fluorescence (red look-up table (LUT)). After 14min, the blue light is switched off, and the ambient red light is placed. Chloroplasts spread out within a larger time scale. Note the adjusted time step.

Movie S7. Network analysis of chloroplast autofluorescence signal. Nodes (dots) between the edges of the skeletonized image are tracked over time. The dataset corresponds to Fig. 3 and Movie S4.

Movie S8. Peripheral stimulation 488 nm-laser (white box). Chloroplast autofluorescence (red 107 LUT).

Movie S9. Sequential peripheral stimulation 488 nm-laser (white box). Chloroplast autofluorescence (red LUT). Chloroplast shrinks first on the upper stimulation side while simultaneously, the lower side reacts. Then, a second stimulus is applied to the lower side.

Movie S10. Central stimulation with 488 nm-laser (white box). Chloroplast autofluorescence (red LUT).

## Data Availability

Microscope data have been deposited in Zenodo (10.5281/zenodo.13959904) (79).
