# Supplementary material for: Light-regulated chloroplast morphodynamics in a single-celled dinoflagellate
Source: Proc Natl Acad Sci U S A. 2024 Nov 15;121(47):e2411725121. doi: 10.1073/pnas.2411725121 (PMC11588079; doi:10.1073/pnas.2411725121)
Supplement: Supplementary file 1 — Appendix 01 (PDF) [file pnas.2411725121.sapp.pdf]

Supporting Information for

Light-regulated chloroplast morphodynamics in a single-celled dinoflagellate

Nico Schramma, Gloria Casas Canales and Maziyar Jalaal

Maziyar Jalaal.
E-mail: m.jalaal@uva.nl

This PDF file includes:

Supporting text
Figs. S1 to S5
Tables S1 to S2
Legends for Movies S1 to S10
SI References

Other supporting materials for this manuscript include the following:

Movies S1 to S10

Supporting Information Text

Overview. In these supplementary texts we describe spectral properties of the white light source (Supplementary Text I, fig. S1 and microscopic images of the dynamic chloroplast contraction (Supplementary Text II,fig. S2). Additionally, we present a detailed description of the mathematical model (Supplementary Text III, fig. S3), its analytical solutions and fitting to experiments fig. S4. Further, we describe pharmacological perturbations (Supplementary Text IV, S5) to the chloroplast motion by drugs targeting cytoskeletal components and molecular motors.

Supplementary Text I: Spectral properties of the white light source. We perform white light stimulation experiments (Fig. 1) to investigate the response of *Pyrocystis lunula* to light. To compare the light conditions with the natural light conditions of the organism we measure the lamp spectrum using a spectrometer and compare it to measured spectra in the euphotic zone in various oceans (fig. S1), data obtained from Morel et al. (2007) (1) and Holtrop et al. (2020) (2). Notably the lamp spectra coincide well in the blue light regime in the ocean, however partly lacking the green light spectra. The blue light peak is also in line with the blue light sensors phototropin (3). The lamp light irradiance $I = 7.6\,\mathrm{mW/cm^2}$ also models well the overall light intensity in the approximately $50 - 100\,\mathrm{m}$-deep euphotic zone.

Supplementary Text II: Dynamic chloroplast contraction. We present the irradiance-dependent chloroplast contraction in fig. S2. The imaging corresponds to lines of individual experiments in the main text figure 1C,D. The chloroplasts contract transiently in weak light ($I = 0.6\,\mathrm{mW/cm^2}$) fig. S2A. Under dim light they do not fully contract toward the cell's center (fig. S2B), while they fully contract toward the center under strong light ($I = 41\,\mathrm{mW/cm^2}$).

Supplementary Text III: Active Kelvin-Voigt model.

Model overview. We model the light-induced chloroplast contraction and expansion. For that we assume a one-directional (uni-axial) contraction and expansion of the chloroplast material. This assumption is based on the observation of negligible expansion or contraction in perpendicular directions during the photo-response (Fig. 2A,B,D, Movie S4-S6). The relative contraction can be described by the spatial variable $x(t)$, which relates to the normalized area by: $\mathcal{A}/\mathcal{A}_0 = 1 - x$, i.e. $x = 0$ if the chloroplast is fully extended.
The forces responsible for this movement are imposed along the chloroplast network. This assumption is supported by the observed structural similarity between actin-network and chloroplast structures (4). These actin-mediated forces result in a new target position $p(t)$ for the chloroplast and are triggered by a chemical signal, which is the product of light sensation by light receptors in the cell.
The absence of the contractile force leading to $p = 0$, will result in a relaxation of the chloroplast into extended configuration ($x = 0$), while forcing, i.e. $p > 0$, will cause the chloroplast to contract (fig. S3C,D). The response in either direction (active contraction/expansion) occurs with two time scales τ_{KV} and τ^*_{KV} summarizing active and passive properties such as visco-elastic damping, molecular signaling time scales, turn-over rates, and cannot be ascribed to a unique process.
Experimental measurements indicate that the fluctuations in chloroplast position are small compared to the global motion (Fig. 1B,D, Movie S1-S3). This allows us to neglect noise in this model.

Equations of motion. The stress-induced motion can be formalized as a visco-elastic Kelvin-Voigt solid:

$$\tau_{KV}\frac{\mathrm{d}x(t)}{\mathrm{d}t} + x(t) = p(t). \qquad [1]$$

The new equilibrium position $p(t)$ is a result of active contraction and is controlled by signaling chemicals in the following way (fig. S3A,B): We hypothesize that light is captured by two different light sensors which send chemical signals c_1 and c_2 upon light simulation within the time scales $\tau_{1,2}$, respectively:

$$\tau_{1,2}\frac{\mathrm{d}c_{1,2}(t)}{\mathrm{d}t} + c_{1,2}(t) = s_{1,2}(I(t)), \qquad [2]$$

where $s_{1,2}(I)$ are the respective light-response functions.
The first sensor produces a chemical c_1 leading to a *photo-avoidance* response, which triggers the contraction of the chloroplast material. Here, we assume that the first light sensor will operate at all intensities $I > 0$, such that that $s_1(I) = \alpha_1 I$ with $\alpha_1 > 0$ a constant.
The second sensor produces a chemical c_2 suppressing the *photo-avoidance* response, i.e., inhibiting the contraction of the chloroplast. The second sensor only operates at weak intensities below a threshold $0 < I < I_{th}$. Thus the response function reduces to $s_2(I) = \alpha_2(H(I_{th} - I))$, with a step function H such that: $s_2(I) = 0$ if $I \geq I_{th}$ or $I = 0$, and $s_2(I) = \alpha_2 I$ if $0 < I < I_{th}$. The opposing signals result in the forced position $p(t)$, which we model to depend linearly on c_1 and c_2: $p(t) = \beta(c_1(t) - c_2(t))$. For simplicity we require $\alpha_1 = \alpha_2 = \alpha$ such that $\Delta c(t \to \infty) = 0$, resulting in a full relaxation $x(t \to \infty) = 0$ during the transient response under constant weak light.
Next, we will present the solution in the cases of constant high light $I \geq I_{th}$ and low light $I < I_{th}$, which are turned on at $t = 0$.

Solution for constant strong light $I \geq I_{th}$. Upon constant strong light $I > I_{th}$ we can solve equations Eq. (1) and Eq. (2) with initial conditions $c_1 = 0,\ x = 0$ (fig. S3A,C). These equations are solved analytically for constant light irradiation starting at $t = 0$ (as in our experiments Fig. 1):

$$x(t) = \alpha\beta I \left[1 - e^{-t/\tau_{KV}} + \frac{\tau_1}{\tau_{KV} - \tau_1}\left(e^{-t/\tau_1} - e^{-t/\tau_{KV}}\right)\right] \qquad [3]$$

We combine α, β and I into a single fit parameter: $\Delta\mathcal{A}_{max} = \alpha\beta I$, representing the maximal contraction reached for $t \to \infty$.

Solution for transient response at constant low light $I < I_{th}$. At low light intensities, we observe a transient response (Fig. 1D,fig. S3B,D). Below a threshold intensity ($0 < I < I_{th}$), a second light sensor sends a suppressive signal counteracting the contractile signal of c_1 (fig. S3), similar to models of light adaptation in green algae (5). For times greater than $T_{max} = argmax(x)$, the direction of chloroplast motion reverses (expansion, $\frac{\lceil x}{\lceil \sqcup} < 0$). As the time scale of chloroplast extension τ^*_{KV} is found to be different from that of chloroplast contraction τ_{KV}, equation Eq. (1) will then follow a slower contractile response. The analytical solution for the case of $\tau_{KV} = \tau^*_{KV}$ reads:

$$x(t) = \alpha\beta I \left[\frac{\tau_2}{\tau_2 - \tau_{KV}}\left(e^{-t/\tau_2} - e^{-t/\tau_{KV}}\right) + \frac{\tau_1}{\tau_{KV} - \tau_1}\left(e^{-t/\tau_1} - e^{-t/\tau_{KV}}\right)\right] \qquad [4]$$

Dynamic filter-properties of chloroplast response. The observed behavior of *P. lunula* crucially facilitates the optimization of photosynthesis while minimizing photo-damage. We interpret such a response as a result of the cells signal-processing mechanism upon light sensing. Assuming there is a cost to such a response, relevant and irrelevant light fluctuations have to be discriminated. Consequently, our model can be viewed from the perspective of linear filters (if $I > I_{th}$). We can easily see that the super-threshold dynamic equations will restore a harmonic oscillator by inserting equation Eq. (1) into Eq. (2) with $f = \beta c_1$ (assuming $\tau_{KV} = \tau^*_{KV}$):

$$\frac{\mathrm{d}^2 x}{\mathrm{d}t^2} + \frac{\tau_1 + \tau_{KV}}{\tau_1 \tau_{KV}}\frac{\mathrm{d}x}{\mathrm{d}t} + \frac{x}{\tau_1 \tau_{KV}} = \alpha\beta I(t) \qquad [5]$$

For any combination of the τ_{KV} and τ_1 this system is over-damped as $0 < \frac{(\tau_1+\tau_{KV})^2}{4(\tau_1\tau_{KV})^2} - \frac{1}{\tau_1\tau_{KV}}$. If our light-input is Fourier-transformable, i.e. $\hat{I}(\omega) = \int_{-\infty}^{\infty} I(t)\, e^{-i\omega t}\mathrm{d}t$ exists, we can find a linear response relationship $\hat{x}(\omega) = \hat{\xi}(\omega)\hat{I}(\omega)$, with a susceptibility $\xi(\omega) = \alpha\beta\left((1 - (\tau_1 + \tau_{KV})i\omega)(\tau_1\tau_{KV})^{-1} - \omega^2\right)^{-1}$. The absolute susceptibility

$$|\xi(\omega)| = \frac{\alpha\beta}{\sqrt{(1 + \tau_{KV}^2\omega^2)(1 + \tau_1^2\omega^2)}} \qquad [6]$$

quantifies the filtering capabilities showing that the system essentially behaves similar to a low-pass Butterworth filter (6), cutting off high frequencies $\omega \gg 2\pi/\tau_1 \approx 3.5\,\mathrm{min}^{-1}$, i.e. noisy environmental fluctuations, and adapting towards slow trends of light exceeding time scales of $\tau \gg \tau_{KV} \approx 2.5 - 5\,\mathrm{min}$, i.e. $\omega \ll 1.25 - 2.5\,\mathrm{min}^{-1}$ (fig. S3E). The cut-off frequency for a $-3\,\mathrm{dB}$ signal attenuation, which corresponds to the power-ratio $|\xi(\omega)|^2/|\xi(0)|^2 = 0.5$, is $\omega_0 \approx 0.32\,\mathrm{min}^{-1}$, relating to a time scale of approximately 3 minutes.

Model fitting. We fit the model's solutions Eq. (4) and Eq. (3) to the averages of multiple experiments at a given light value and numerically integrated equations Eq. (1) and Eq. (2) for periodic stimulation (Fig. 1D, Fig. S4A-D, Table S1). We use the dogbox method with a Huber loss function. For periodic illumination experiments, the R^2 value is decreased because of out-of-focus imaging over long times, and therefore, the expansion area was underestimated. Additionally, we fitted each experiment individually (Fig. S4E) to test the robustness of the model. The fitting parameters remained notably consistent for all different experiments and physiological conditions (see Table S1).

Supplementary Text IV: Pharmacological treatment. Cells were treated with $5\,\mu$M Nocodazole (microtubule depolimerization), $10\,\mu$M Latrunculin B (actin depolimeization) or 2 mM BDM (myosin inhibition), all prepared in aqueous f/2 solution. Prior to treatment, cells were adapted to either darkness or bright light conditions ($I \approx 30\,\mathrm{mW/cm^2}$). Following the treatments, the cells were incubated for another 2 h in darkness or bright light conditions ($I \approx 30\,\mathrm{mW/cm^2}$). Microscopy is performed simultaneously for all treatment groups: first, dark- and light-adapted cells are subjected to darkness for 70 min. This was followed by exposure to intermediate light levels, with a subsequent increase in light intensity after 30 min.
To measure the effect, we count the cells and measure the overall increase or decrease of the average pixel values with respect to the first time point. The data is normalized for the number of cells in the field of view to account for size effects.
The unperturbed control-group of light adapted cells ($N = 1588$) expands at dim light conditions within 50 min (fig. S5A,B). BDM ($N = 1138$) and Nocodazole ($N = 1239$) treated cells do not change and expand as efficiently as the control group. Latrunculin B-treated cells ($N = 1724$) do not expand their chloroplasts, as confirmed by the vanishing slope of the relative normalized absorbance (fig. S5A) and by visual inspection (fig. S5B).
For the group of dark adapted cells, which are subjected to intermediate light intensities (Time ≤ 30 min) and strong light intensities (Time > 30 min), we find that all groups but the Latrunculin-B treated cells ($N = 1724$) adapt to the strong light stimulus, as confirmed by the decline in absorbance and visual inspection (fig. S5C,D). Our pharmacological inhibitions clearly suggest that the actin network is used both in chloroplast contraction and expansion. The role of myosin and microtubules is not fully clear and might be concentration dependent, as suggested in studies on the diurnal chloroplast motion (4).

SI Figures

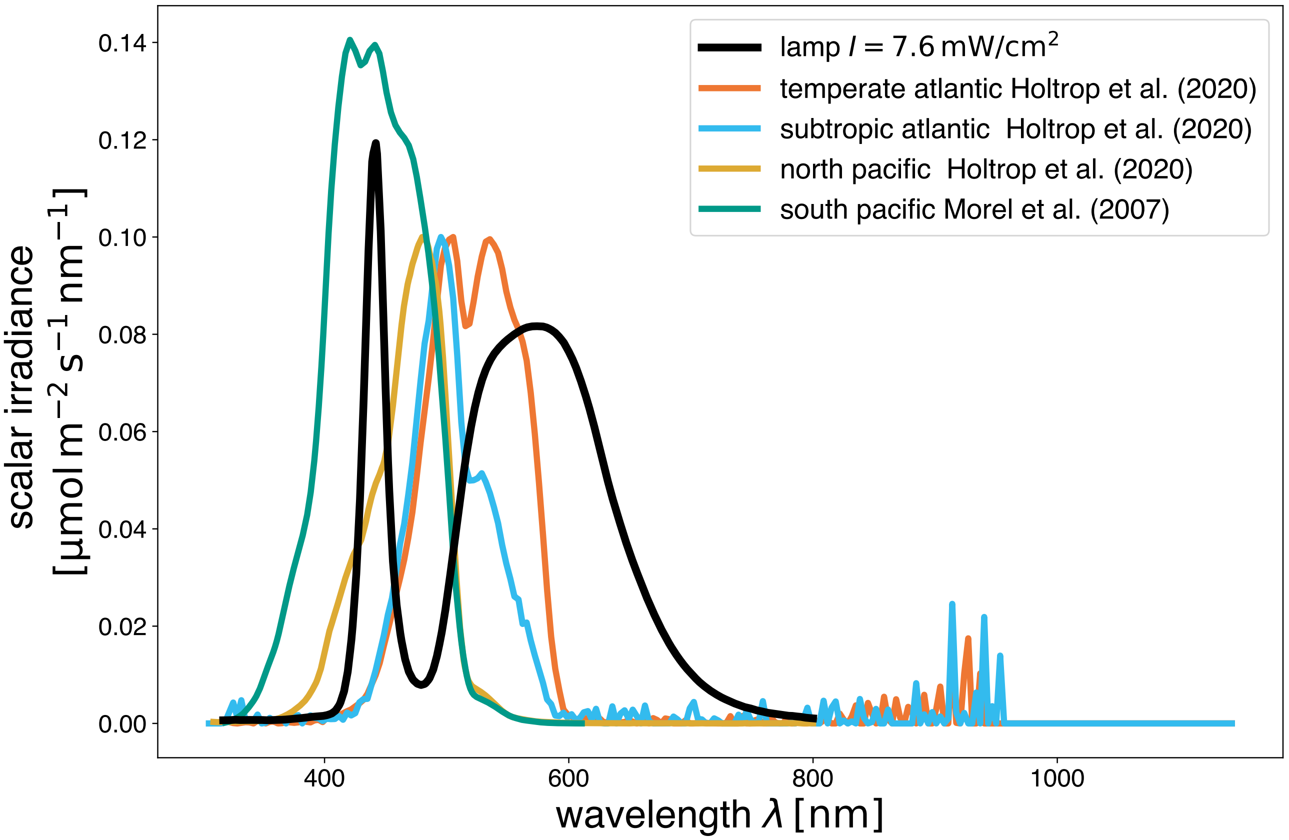

Fig. S1. Measured spectrum of the lamp at $I = 7.9\,\mathrm{mW/cm^2}$ (black) in comparison to the light spectra in the euphotic depth (colored lines). The spectrum suggests that the green light is reduced compared to aquatic environments, while blue and red spectra, on average, overlap the oceanic light conditions. Measured data in the euphotic zone in the North Pacific Ocean and Atlantic from (2). Data for South Pacific from (1).

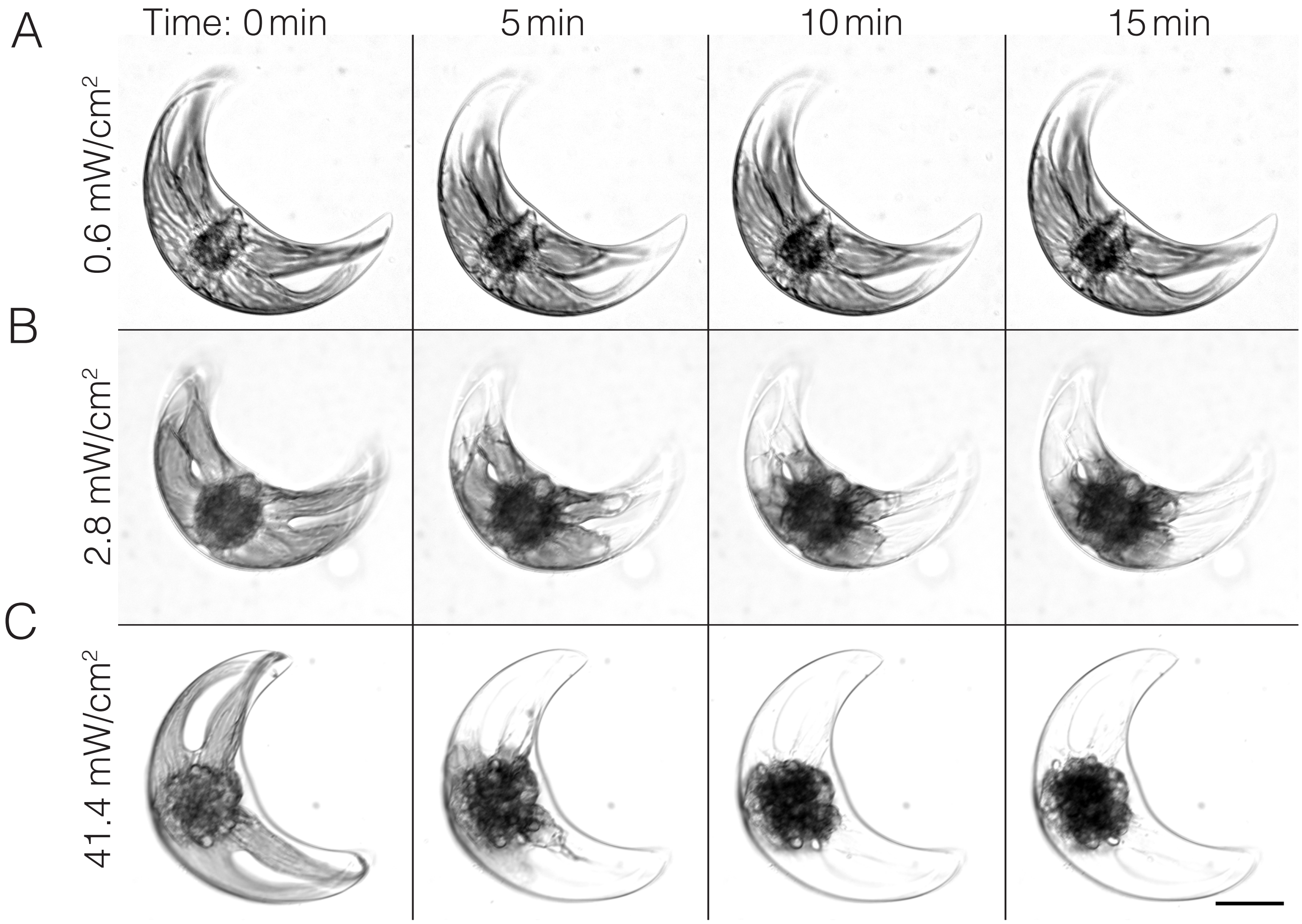

Fig. S2. Irradiance-dependent chloroplast retraction. (**A**) at low intensities, chloroplast contraction is limited to a transient response. (**B**) Intermediate intensities lead to an incomplete retraction of the chloroplast. (**C**) High-intensity light stimulation leads to a complete retraction of the chloroplast towards the cytoplasmic core area. Scale bar: $30\,\mu\mathrm{m}$

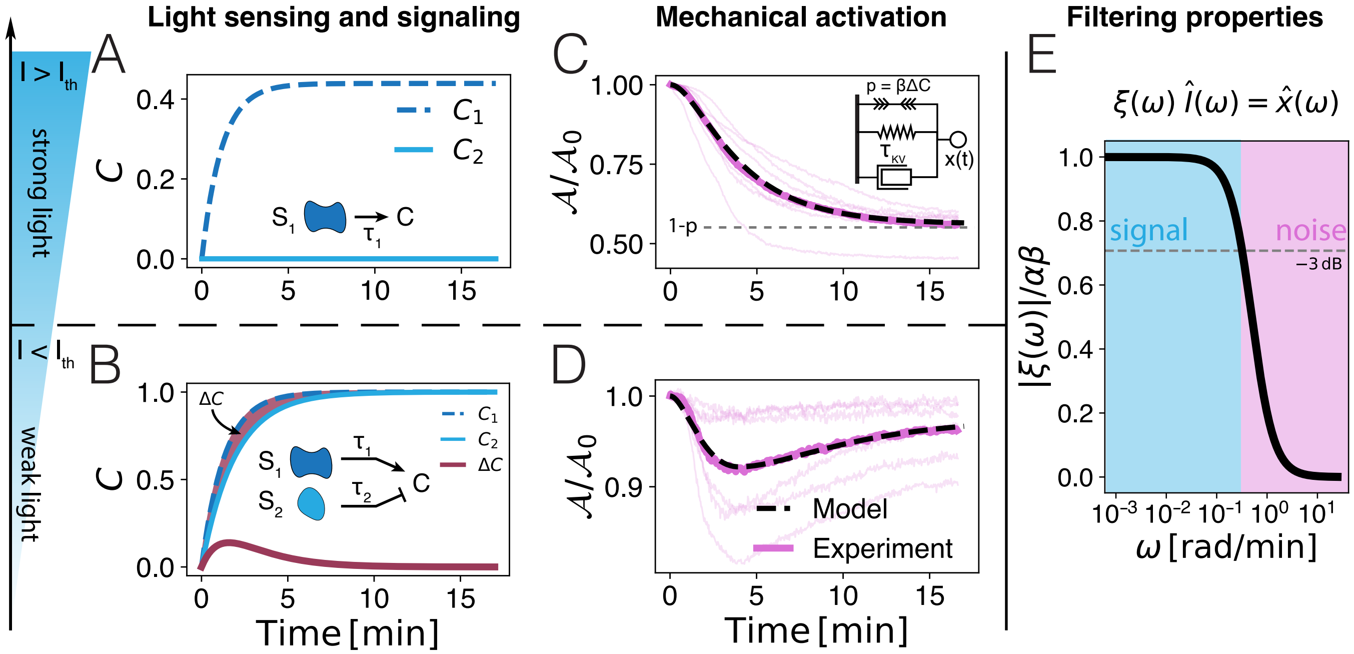

Fig. S3. Schematic representation of irradiance-dependent signaling model. Left: Light sensing by two hypothetical light sensors (S_1 and S_2) leads to an intensity-dependent response of signalling molecules c_1 (dotted, dark blue) and c_2 (solid line, light blue). Above a light threshold $I > I_{th}$ (top) only c_1 is increased, while below this threshold a combination of two opposing signaling molecules is released with two time scales $\tau_1 < \tau_2$ (bottom). This leads to a non-monotonic curve of the concentration ΔC (magenta line). Insets depict signalling model. The concentration difference triggers a contractile stress f (inset, center) for a viscoelastic solid medium (Kelvin-Voigt element) with two relaxation timescales τ_{KV} (compression) and τ^*_{KV} (extension). The area reduction of the chloroplast is $\mathcal{A}/\mathcal{A}_0 = 1 - x$. This model (black dashed lines) fits the observation (purple). Thin purple lines: individual experiments. The response x depending on I can be measured in terms of a susceptibility (linear response function) in Fourier space $\xi(\omega) = \hat{x}(\omega)/\hat{I}(\omega)$. The amplitude quantifies the filtering properties: high-frequency frequencies (noise) are dampened, while slow variations (signal) can be adapted to. The dotted line represents the $-3\,\mathrm{dB}$ line at which the power $|\xi(\omega)|^2$ is half.

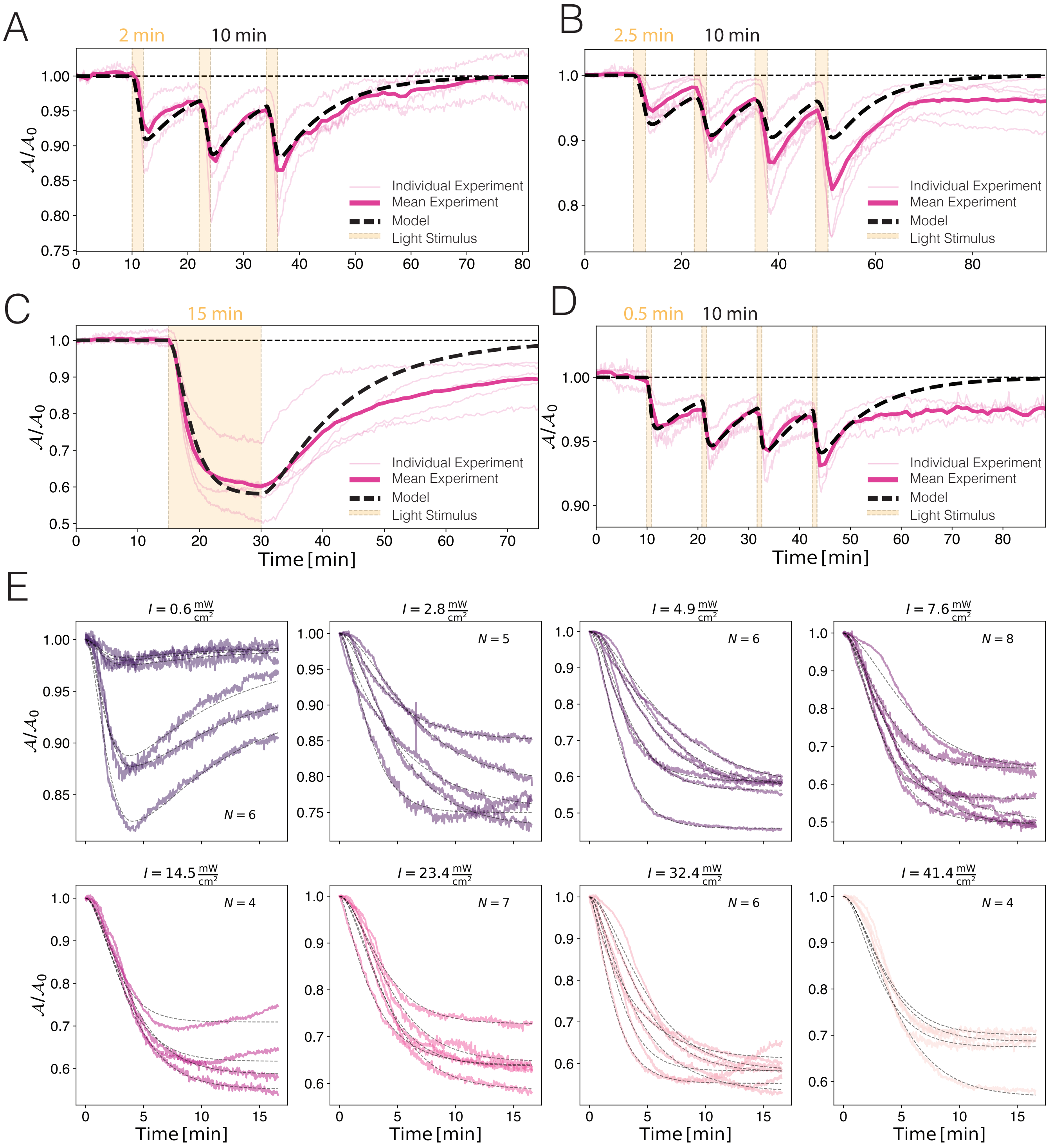

Fig. S4. (A-D) Integration of dynamical model (for $I > I_{th}$) (dotted line) compared to experimental data (violet: mean, thin lines: individual experiments) for different durations of alternating white light illumination (orange regions) and dim red light. Parameters are given in Table S1. At long times, the area was underestimated due to out-of-focus effects. (E) Fits of all individual experiments at different light irradiances (colors). Fit parameters are given in Table S2.

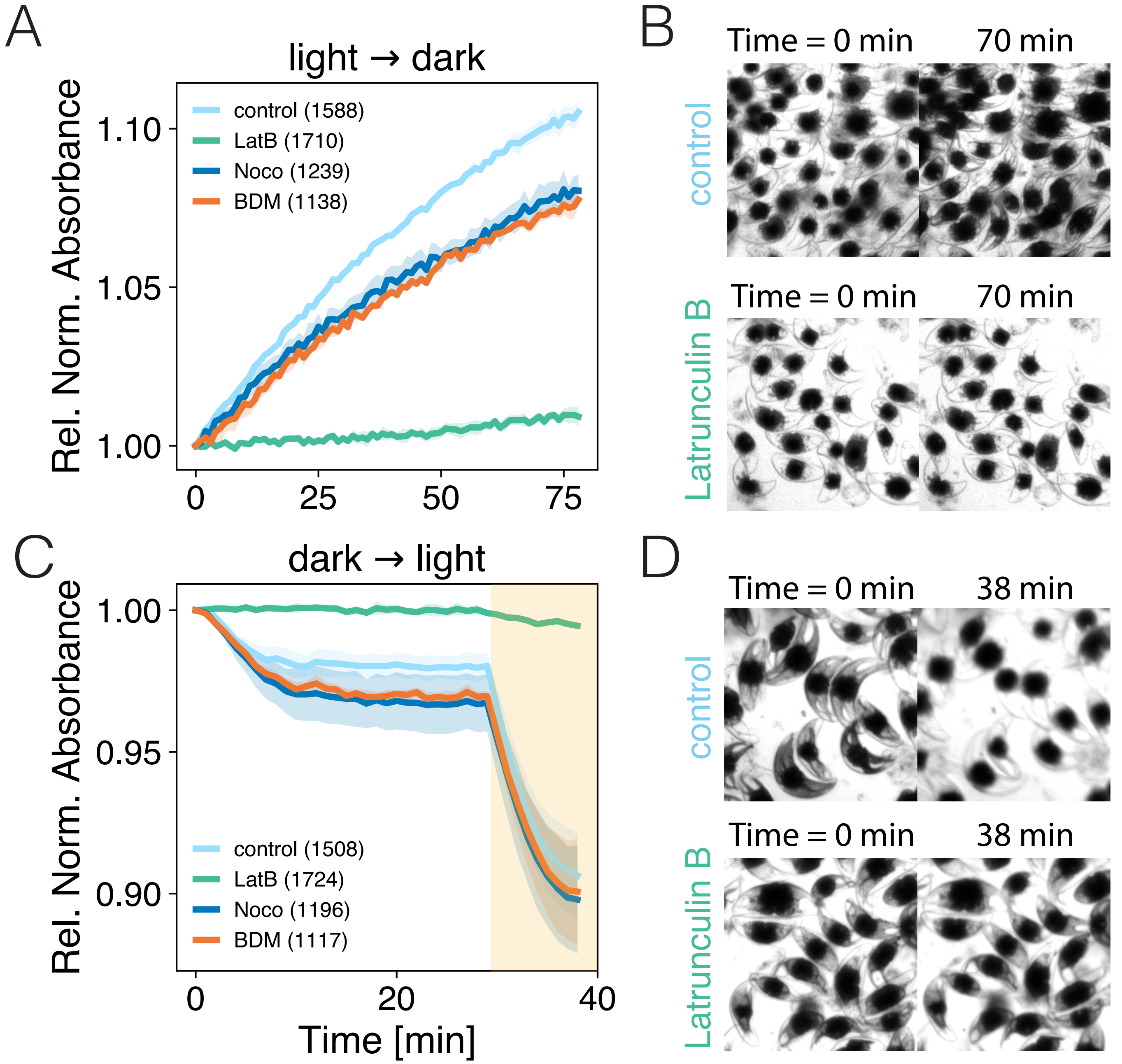

Fig. S5. Analysis of pharmacological perturbation to the photoadaptation mechanism. (**A**) Light-adapted cells are placed in dark light conditions and spread out (control). Absorption is measured and normalized with the number of cells per experiment. $10\,\mu\mathrm{M}$ Latrunculin B treated cells (LatB) do not react. Both $5\,\mu\mathrm{M}$ Nocodazole (Noco) and $2\,\mathrm{mM}$ 2,3-butanedione monoxime (BDM) do not show a significant effect. Numbers in the legend stand for the total amount of cells per treatment. Shadowed regions display the min-max error of two independent batches. (**B**) Example images for control and Latrunculin B treated cells before and after dark adaptation. (**C**) Absorption measurement of dim light-adapted cells placed in bright light, with subsequent increase of light intensity (shaded area). (**D**) Example for control and Latrunculin B treated cells before and after bright light adaptation.

**Figure**	**Irradiance** $[\mathrm{mW/cm^2}]$	τ_1 [min]	τ_2 [min]	τ_{KV} [min]	τ^*_{KV} [min]	$\Delta\mathcal{A}_{max}$	R^2
Fig. 1B, S4A	2.8	0.533 ± 0.235	–	4.153 ± 0.862	9.419 ± 1.031	0.295 ± 0.038	0.928
Fig. S4B	4.9	0.772 ± 0.289	–	6.755 ± 2.606	9.741 ± 1.71	0.291 ± 0.058	0.623
Fig. S4C	4.9	1.826 ± 0.345	–	2.039 ± 0.526	12.907 ± 1.37	0.421 ± 0.01	0.832
Fig. S4D	4.9	0.62 ± 0.172	–	4.265 ± 0.848	10.43 ± 0.931	0.335 ± 0.026	0.211
Fig. 1D	0.6	1.324 ± 0.4	1.936 ± 0.357	3.107 ± 1.047	5.997 ± 0.932	1.0 ± 1.042	0.976
Fig. 1D	2.8	0.788 ± 0.015	–	4.032 ± 0.029	–	$0.223 \pm 3 \cdot 10^{-4}$	0.999
Fig. 1D	4.9	1.157 ± 0.024	–	3.346 ± 0.038	–	$0.443 \pm 5 \cdot 10^{-4}$	0.999
Fig. 1D	7.6	1.772 ± 0.074	–	2.492 ± 0.089	–	$0.44 \pm 6 \cdot 10^{-4}$	0.999
Fig. 1D	14.5	1.887 ± 0.044	–	1.877 ± 0.039	–	0.39 ± 0.001	0.991
Fig. 1D	23.4	2.003 ± 0.012	–	1.993 ± 0.014	–	$0.35 \pm 5 \cdot 10^{-4}$	0.996
Fig. 1D	32.4	1.042 ± 0.025	–	2.603 ± 0.03	–	$0.421 \pm 3 \cdot 10^{-4}$	0.999
Fig. 1D	41.4	2.009 ± 0.054	–	1.999 ± 0.042	–	0.343 ± 0.001	0.981
Fig. 3G	3	$1.471 \pm 4.6 \cdot 10^{-4}$	-	$1.472 \pm 1.8 \cdot 10^{-4}$	-	$0.648 \pm 1 \cdot 10^{-4}$	0.999
Fig. 4C top	3.25	2.595 ± 2.426	3.952 ± 7.448	2.531 ± 2.93	$= \tau_{KV}$	1.0 ± 7.124	0.88
Fig. 4C bottom	3.25	2.021 ± 0.178	–	2.012 ± 0.258	–	0.529 ± 0.016	0.982
Fig. 4D top	3.25	1.951 ± 0.046	–	1.942 ± 0.065	–	0.799 ± 0.006	0.999
Fig. 4D bottom	3.25	2.244 ± 0.061	–	2.236 ± 0.095	–	0.729 ± 0.008	0.998

Table S1. Fit parameters for the mathematical model (Supplementary Text 2). With the referred figure, applied lamp irradiance, and fitted parameters τ_1,τ_2,τ_{KV},τ^*_{KV} and $\Delta\mathcal{A}_{max}$. Note: small R^2 in Figure S4D due to out-of focus effects in data, such that area never reaches $\mathcal{A} = 1$. During transient response (Fig. 1D and 4C top) $\Delta\mathcal{A}_{max}$ can exceed 1.

**Irradiance** $[\mathrm{mW/cm^2}]$	τ_1 [min]	τ_2 [min]	τ_{KV} [min]	τ^*_{KV} [min]	$\Delta\mathcal{A}_{max}$	R^2
0.6	1.039 ± 1.398	1.456 ± 1.604	3.092 ± 5.214	6.97 ± 5.269	0.405 ± 2.549	0.381
0.6	1.315 ± 0.445	2.135 ± 0.34	2.654 ± 0.767	6.726 ± 0.704	1.125 ± 0.937	0.978
0.6	1.112 ± 0.203	1.759 ± 0.281	4.253 ± 1.327	8.019 ± 1.206	2.366 ± 1.491	0.976
0.6	0.909 ± 0.661	1.198 ± 1.048	6.732 ± 4.054	8.287 ± 2.958	4.048 ± 24.867	0.91
0.6	1.029 ± 1.376	1.307 ± 1.693	3.104 ± 4.131	6.904 ± 4.219	0.479 ± 5.0	0.599
0.6	1.144 ± 3.258	1.4 ± 2.976	2.541 ± 4.962	6.336 ± 5.085	0.417 ± 9.671	0.574
2.8	0.1 ± 0.035	–	5.105 ± 0.094	–	0.248 ± 0.001	0.982
2.8	1.499 ± 0.062	–	3.792 ± 0.101	–	$0.271 \pm 9 \cdot 10^{-4}$	0.997
2.8	1.059 ± 0.044	–	3.578 ± 0.074	–	$0.149 \pm 4 \cdot 10^{-4}$	0.995
2.8	1.381 ± 0.016	–	1.39 ± 0.018	–	$0.25 \pm 5 \cdot 10^{-4}$	0.987
2.8	3.394 ± 0.018	–	3.385 ± 0.034	–	$0.208 \pm 7 \cdot 10^{-4}$	0.996
4.9	1.846 ± 0.016	–	1.836 ± 0.02	–	$0.438 \pm 9 \cdot 10^{-4}$	0.993
4.9	1.806 ± 0.094	–	5.722 ± 0.213	–	0.436 ± 0.004	0.996
4.9	2.571 ± 0.009	–	2.563 ± 0.015	–	$0.424 \pm 6 \cdot 10^{-4}$	0.998
4.9	1.18 ± 0.065	–	2.042 ± 0.078	–	$0.413 \pm 7 \cdot 10^{-4}$	0.995
4.9	2.182 ± 0.162	–	3.819 ± 0.23	–	0.431 ± 0.002	0.995
4.9	1.034 ± 0.034	–	1.718 ± 0.04	–	$0.542 \pm 4 \cdot 10^{-4}$	0.998
7.6	3.006 ± 0.035	–	2.998 ± 0.062	–	0.362 ± 0.002	0.982
7.6	1.827 ± 0.015	–	1.817 ± 0.019	–	$0.502 \pm 10 \cdot 10^{-4}$	0.993
7.6	1.491 ± 0.047	–	3.23 ± 0.067	–	0.511 ± 0.001	0.998
7.6	1.572 ± 0.008	–	1.573 ± 0.009	–	$0.437 \pm 4 \cdot 10^{-4}$	0.997
7.6	0.734 ± 0.029	–	4.44 ± 0.063	–	0.502 ± 0.001	0.996
7.6	1.42 ± 0.106	–	2.426 ± 0.131	–	$0.359 \pm 10 \cdot 10^{-4}$	0.993
14.5	1.422 ± 0.028	–	1.412 ± 0.033	–	0.291 ± 0.001	0.963
14.5	2.077 ± 0.019	–	2.07 ± 0.025	–	0.413 ± 0.001	0.991
14.5	1.751 ± 9.217	–	1.77 ± 9.254	–	0.383 ± 0.002	0.976
14.5	2.034 ± 0.012	–	2.027 ± 0.015	–	$0.448 \pm 7 \cdot 10^{-4}$	0.996
23.4	0.33 ± 0.015	–	2.805 ± 0.026	–	$0.361 \pm 5 \cdot 10^{-4}$	0.996
23.4	1.946 ± 0.018	–	1.939 ± 0.024	–	$0.363 \pm 9 \cdot 10^{-4}$	0.991
23.4	2.343 ± 0.023	–	2.334 ± 0.033	–	0.353 ± 0.001	0.989
23.4	2.237 ± 0.019	–	2.227 ± 0.027	–	0.413 ± 0.001	0.992
23.4	2.032 ± 0.021	–	2.023 ± 0.028	–	$0.272 \pm 8 \cdot 10^{-4}$	0.988
32.4	0.525 ± 0.017	–	1.733 ± 0.023	–	$0.447 \pm 4 \cdot 10^{-4}$	0.996
32.4	2.31 ± 0.008	–	2.304 ± 0.011	–	$0.421 \pm 5 \cdot 10^{-4}$	0.998
32.4	0.1 ± 0.017	–	3.536 ± 0.035	–	$0.389 \pm 8 \cdot 10^{-4}$	0.993
32.4	2.631 ± 0.028	–	2.621 ± 0.044	–	0.416 ± 0.002	0.986
32.4	1.403 ± 0.035	–	1.394 ± 0.041	–	0.417 ± 0.002	0.943
32.4	0.686 ± 0.022	–	3.657 ± 0.041	–	$0.469 \pm 9 \cdot 10^{-4}$	0.997
41.4	2.406 ± 0.017	–	2.397 ± 0.025	–	0.433 ± 0.001	0.994
41.4	1.992 ± 0.028	–	1.984 ± 0.037	–	0.313 ± 0.001	0.98
41.4	1.97 ± 0.041	–	1.962 ± 0.054	–	0.299 ± 0.002	0.96
41.4	1.733 ± 0.031	–	1.728 ± 0.039	–	0.325 ± 0.001	0.971

Table S2. Fit parameters for all curves in Figure S4E. τ_1,τ_2,τ_{KV},τ^*_{KV} **and** $\Delta\mathcal{A}_{max}$.

SI Movies

Movie S1. Movie S1: Chloroplast contraction of *P. lunula* at different light intensities: (left) $0.6\,\mathrm{mW/cm^2}$**, (center)** $2.8\,\mathrm{mW/cm^2}$ **and (right)** $41.4\,\mathrm{mW/cm^2}$**, corresponding to S2A-C, respectively.**

Movie S2. Movie S2: Dynamic light-controlled chloroplast motion with $10\,\mathrm{min}$ $0.4\,\mathrm{mW/cm^2}$ **red-light imaging between** $2.5\,\mathrm{min}$**-lasting white-light stimulation at** $I = 7.6\,\mathrm{mW/cm^2}$**.**

Movie S3. Movie S3: Dynamic light-controlled chloroplast motion with $15\,\mathrm{min}$ $0.4\,\mathrm{mW/cm^2}$ **red-light imaging between** $15\,\mathrm{min}$**-lasting white-light stimulation at** $I = 7.6\,\mathrm{mW/cm^2}$**.**

Movie S4. Movie S4: Time series for global stimulation with blue light ($470 \pm 50\,\mathrm{nm}$**. Imaging of chlorophyll auto-fluorescence (red look-up table (LUT)).**

Movie S5. Movie S5: Time series for a second global stimulation with blue light ($470 \pm 50\,\mathrm{nm}$**. Imaging of chlorophyll auto-fluorescence (red look-up table (LUT)). The sequential buckling of cytoplasmic strands is clearly visible.**

Movie S6. Movie S6: Time series for a global stimulation with blue light ($470 \pm 50\,\mathrm{nm}$**. Imaging of chlorophyll auto-fluorescence (red look-up table (LUT)). After** $14\,\mathrm{min}$**, the blue light is switched off, and the ambient red light is placed. Chloroplasts spread out within a larger time scale. Note the adjusted time step.**

Movie S7. Movie S7: Network analysis of chloroplast autofluorescence signal. Nodes (dots) between the edges of the skeletonized image are tracked over time. The dataset corresponds to Fig. 3 and Movie S4.

Movie S8. Movie S8: Peripheral stimulation $488\,\mathrm{nm}$**-laser (white box). Chloroplast autofluorescence (red LUT).**

Movie S9. Movie S9: Sequential peripheral stimulation $488\,\mathrm{nm}$**-laser (white box). Chloroplast autofluorescence (red LUT). Chloroplast shrinks first on the upper stimulation side while simultaneously, the lower side reacts. Then, a second stimulus is applied to the lower side.**

Movie S10. Movie S10: Central stimulation with $488\,\mathrm{nm}$**-laser (white box). Chloroplast autofluorescence (red LUT).**

References

1. A Morel, et al., Optical properties of the "clearest" natural waters. *Limnol. Oceanogr.* **52**, 217–229 (2007).
2. T Holtrop, et al., Vibrational modes of water predict spectral niches for photosynthesis in lakes and oceans. *Nat. Ecol. Evol.* **5**, 55–66 (2021).
3. WR Briggs, JM Christie, Phototropins 1 and 2: Versatile plant blue-light receptors. *Trends Plant Sci.* **7**, 204–210 (2002).
4. K Heimann, PL Klerks, KH Hasenstein, Involvement of actin and microtubules in regulation of bioluminescence and translocation of chloroplasts in the dinoflagellate Pyrocystis lunula. *Bot. Mar.* **52**, 170–177 (2009).
5. K Drescher, RE Goldstein, I Tuval, Fidelity of adaptive phototaxis. *Proc. Natl. Acad. Sci. United States Am.* **2010** (2010).
6. S Butterworth, , et al., On the theory of filter amplifiers. *Wirel. Eng.* **7**, 536–541 (1930).
